# *In vivo* real-time assessment of developmental defects in enamel of anti-Act1 mice using optical coherence tomography

**DOI:** 10.1016/j.heliyon.2023.e16545

**Published:** 2023-05-22

**Authors:** Sujuan Zeng, Yuejun Wu, Lijing Wang, Yuhang Huang, Wenyan Huang, Ziling Li, Weijian Gao, Siqing Jiang, Lihong Ge, Jian Zhang

**Affiliations:** a Department of Pedodontics, Affiliated Stomatology Hospital of Guangzhou Medical University, Guangdong Engineering Research Center of Oral Restoration and Reconstruction, Guangzhou Key Laboratory of Basic and Applied Research of Oral Regenerative Medicine, Guangzhou, 510182, China; b Vascular Biology Research Institute, Guangdong Pharmaceutical University, Guangzhou, 510006, China; c School of Biomedical Engineering, The Sixth Affiliated Hospital of Guangzhou Medical University, Qingyuan People's Hospital, Guangzhou Medical University, Guangzhou, 511436, China; d Department of Temporomandibular Joint, Affiliated Stomatology Hospital of Guangzhou Medical University, Guangzhou Key Laboratory of Basic and Applied Research of Oral Regenerative Medicine, Guangzhou, 510182, China; e Department of Pediatric Dentistry, Stomatology Hospital of Peking University, Beijing, 100081, China

**Keywords:** OCT, Enamel development, Enamel defect

## Abstract

The purpose of this study was to explore the feasibility of using optical coherence tomography (OCT) for real-time and quantitative monitoring of enamel development in gene-edited enamel defect mice. NF-κB activator 1, known as Act1, is associated with many inflammatory diseases. The antisense oligonucleotide of Act1 was inserted after the CD68 gene promoter, which would cover the start region of the Act1 gene and inhibit its transcription. Anti-Act1 mice, gene-edited mice, were successfully constructed and demonstrated amelogenesis imperfecta by scanning electron microscope (SEM) and energy dispersive X-ray (EDX) spectroscopy. Wild-type (WT) mice were used as the control group in this study. WT mice and anti-Act1 mice at 3 weeks old were examined by OCT every week and killed at eight weeks old. Their mandibular bones were dissected and examined by OCT, micro-computed tomography (micro-CT), and SEM. OCT images showed that the outer layer of enamel of anti-Act1 mice was obviously thinner than that of WT mice but no difference in total thickness. When assessing enamel thickness, there was a significant normal linear correlation between these methods. OCT could scan the imperfect developed enamel noninvasively and quickly, providing images of the enamel layers of mouse incisors.

## 1 Introduction

Amelogenesis imperfecta (AI) is an inherited disorder, which affects the structure and appearance of enamel of all teeth [1]. AI is caused by various obstacles during the tooth development process such as tooth matrix formation and matrix calcification. It can lead to permanent defects in tooth hard tissue [2]. The main clinical manifestations of AI are insufficient enamel thickness, poor enamel mineralization, insufficient mineral content, and reduced enamel hardness [3]. Enamel is the hardest tissue of the human body which plays a significant role in bearing chewing pressure in the oral cavity. In addition, it protects the structures of teeth including dentin and fragile dental pulp from external stimulations. AI will not only affects the tooth function of patients, and increases the risk of dental caries, but also affects the appearance, causing certain psychological problems and social disorders [4]. However, we can neither regenerate nor repair enamel.

There are some ways to determine AI. For those with a mild degree, the possible AI can be found by observing the enamel color. But enamel color cannot be reliable evidence because there are still many other tooth discoloration diseases [[5], [6], [7]]. Doctors can also use dental probes to feel the hardness and roughness of the enamel lesion area. Unfortunately, this mechanical operation may directly cause severe damage to the fragile enamel caused by AI. X-ray is also a common diagnostic method. The current classification of enamel hypoplasia is based on Witkop's classification in 1988 [8]. There are four types of AI: Type I Hypoplastic, Type II Hypomaturation, Type III Hypocalcified, and Type IV Hypomaturation-hypoplastic with taurodontism. The X-ray resistivity of enamel contrasts normally with dentin in hypoplastic. In Type II, enamel and dentin are hard to distinguish from each other on radiographs. In Type III and Type IV, enamel shows decreased and increased density relative to dentin respectively. Moreover, a considerable number of teeth with AI only show changes of enamel color which have no difference in X-ray imaging [9]. Therefore, non-invasive and accurate determination of AI becomes an important goal. At the moment, the hand-held digital intraoral scanner has been developed and applied in clinics. It allows dentists quickly scan patients' teeth and obtain dynamic 3D data [10]. The oral scanner can obtain information of the tooth surface, though, the structure beneath the surface cannot be obtained, let alone providing the pathological changes.

Optical coherence tomography (OCT) is a device that detects the delay and intensity of light scattered or reflected from the surface of the tissue. It is a non-contact, non-invasive means of detection that allows tomographic imaging of the internal structure of a certain thickness of tissue without contact examination, without tissue biopsy, and without the need for X-ray examination, obtaining images similar to tissue sections [11]. OCT is a non-radiation imaging diagnostic technique, which has no ionizing radiation and is harmless to the human body. It can be used for chair-side detection in oral clinic and perform a 3D imaging [12]. At present, the fracture of the elderly is more prominent [13,14], and OCT could also be used as a means to detect bone regeneration [15]. OCT detection process is simple, does not need to treat with the tissue to be assessed, does not change the tissue structure, can achieve non-invasive real-time imaging, can monitor tissue changes at any time in clinic, save a lot of clinical operation time and improve the efficiency of treatment. OCT was first used in cardiology, oncology, and ophthalmology [16]. In dentistry, OCT has extremely high application value in the early diagnosis [17,18], treatment assistance [19], and prognosis monitoring [20]. At present, OCT has been used to detect hidden caries, dental microcracks [21], the integrity of tooth filling composite interface [22], early changes of oral malignant tumors, changes of periodontal ligament caused by orthodontic movement, calculus [23] and so on.

NF-κB activator 1, also known as Act1, is mainly expressed in immune cells and modulates their function to regulate inflammation [24]. It is an upstream regulator of IκB kinase and a key mediator of inflammatory and immune response [25]. Macrophages play a vital role in the pathophysiology of inflammatory diseases and Act1 has been proven to be associated with periodontitis, inflammation, and alveolar bone loss. CD68 gene is located on the Chromosome 11 of mouse. Act1 gene is located on the Chromosome 10. Anti-Act1 mice were developed by inserting anti-Act1 antisense oligonucleotides after the CD68 promoter of WT mice causing ultimately inhibition of Act1 gene transcription [26].

In this study, we confirmed initially that anti-Act1 mice can be used as a model of AI. The purpose of this study was to explore the feasibility of using OCT for real-time and quantitative monitoring of enamel development and degree of enamel defect in gene-edited enamel defect mice to simulate the clinic to the maximum extent. The findings may help to develop a testing aid that can evaluate the detect defects in enamel development in a real-time and non-invasive manner, thus allowing clinicians to determine AI more accurately.

## 2 Materials and methods

### 2.1 Animals

**Fig. 1:**
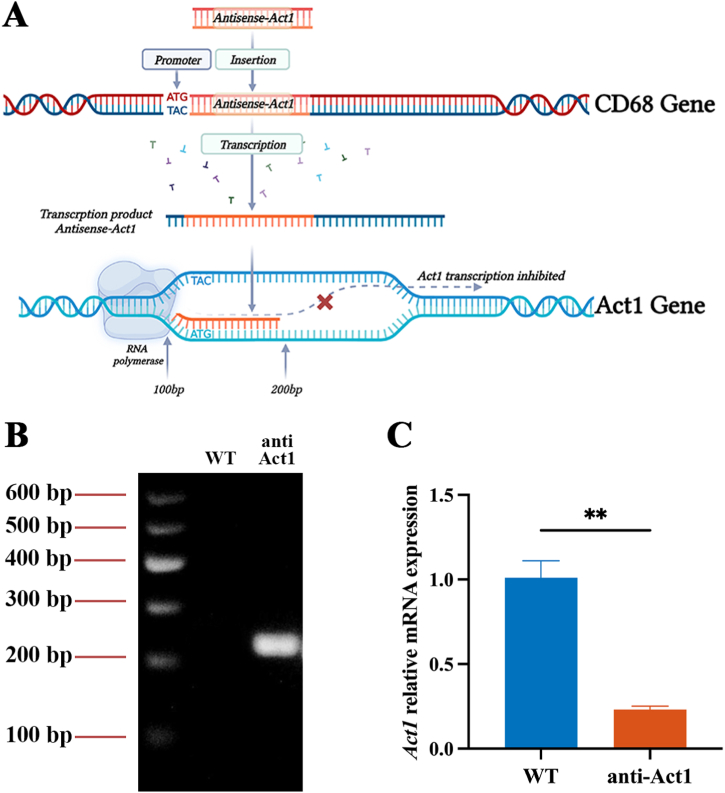
(A) The insertion of antisense on CD68 gene will ultimately inhibit the transcription of Act1 gene. (B) The image of gel separating the PCR product. (C) Act1 gene relative mRNA expression in WT and anti-Act1 mice.

All animal procedures were performed in accordance with the Guidelines for Institutional Animal Care and Use Committee and approved by the Guangdong Laboratory Animals Monitoring Institute (GY2020-001). Wildtype (WT) mice were purchased from Guangdong Medical Animal Experiment Center (Guangdong, China). The anti-Act1 mice used in this study were provided by Lijing Wang's team. Anti-Act1 mice were developed by inserting anti-Act1 antisense oligonucleotides after the CD68 promoter of C57BL/6 mice [26]. After the transcription of CD68 gene, the antisense covered the region from 100 bp prior to ATG start region of Act1 gene to 200 bp posterior to ATG region. This would cause ultimately inhibition of Act1 gene transcription (Fig. 1A). The insertion of antisense was confirmed by PCR using the primer as the following [27]. Forward primer: 5′-CTGGTGCAGACAGCCTAGCTG-3’; reverse primer: 5′-CCTGCGAGCTAAAGTCCTGGA-3’. The PCR product was then separated in agarose gel electrophoresis and visualized by a gel imager (Gel Doc™ XR+, BIO-RAD). The image was shown in Fig. 1B. The Act1 gene expression was detected by RT-qPCR in peritoneal macrophages as shown in Fig. 1C. The primer used for RT-qPCR is showed as the following. Forward: 5′-TCCCGTGGAGGTTGATGAATC-3’; Reverse: 5′-TCAGGGTGCCTTCTAAAGAAACT-3’. Based on the lifelong development, maturation, and eruption of the lower incisors of mice, we chose the lower incisors as the research object. WT mice and anti-Act1 mice of 3–8 weeks old were taken for the experiments. The 3-week-old mice were used in the experiments only after they were weaned considering the animal welfare. They were male with similar weights in healthy conditions. Prior to the experiment, all mice were housed in plastic cages at an ambient temperature of 23–24 °C, maintained on a 12-h diurnal cycle, and received regular feeding.

### 2.2 SEM and EDX spectroscopy analysis

The mandibular bones containing incisors were isolated from WT mice (n = 3) and anti-Act1 mice (n = 3) of 8 weeks old. Each bone was dehydrated with a series of graded ethanol (30 min each of 30%, 50%, 70%, 80%, 90%, 100% × 2), air dried, and fractured at the level of the alveolar crest. The fractured incisors were mounted on metallic stubs using conductive carbon tape and sputter-coated with an Au–Pd film. Samples were imaged at the Laboratory of Stomatology Hospital of Guangzhou Medical University using a HITACHI S–3400 N Scanning Electron Microscope operating at a voltage of 15.0 kV and observed with SEM at 160 and 500 magnifications. The EDX spectroscopy (550i, IXRF Sytems, Austin TX, USA) that was installed on the SEM was used to determine the contents of calcium (Ca, atomic %), phosphorus (P, atomic %), and carbon (C, atomic %) of outer and inner enamel from WT mice and anti-Act1 mice, respectively. Then ratios of calcium and phosphorus (Ca/P) were calculated from each region.

### 2.3 In vivo OCT scanning

The anesthesia machine was used in the experiment. Adjusted the anesthesia concentration of the machine to 3% with 0.5 L/min flow quantity. The induction box was filled with the mixed gas of isoflurane and oxygen. After 1 min, the mice were placed in the induction box and continuously observed. After 2 min, the mice did not move. They were gripped with tweezers and no reaction was observed, indicating that the mice were fully anesthetized. Then the mice were quickly fixed in a supine position on a fixation table and the lower central incisors were exposed to their tooth-alveolar bone junction. The OCT system of the Medical Imaging Innovation Lab was used to record the scan [28] and its schematic was shown as Fig. S1. The OCT system used 840 nm central wavelength (λ), 40 nm bandwidth (Δλ), 5 mW power (P), 12 μm lateral resolution, and 7.24 μm axial resolution. The OCT scanning probe was placed on the mandibular incisors of the mice so that the light emitted by the scanning probe was perpendicular to the labial surface of the mandibular incisors. The scan was performed continuously starting from the incisal end of the incisors, ensuring that the incisal end and the tooth-alveolar bone junction were all within the scan area. The width and length of the scanning image were both 5 mm. Once the scan finished, all mice were awakened and placed back in their cages for further housing. The same scanning procedure was performed on WT mice (n = 6) and anti-Act1 mice (n = 6) at 3, 4, 5, 6, 7, and 8 weeks of age. Both bilateral mandibular incisors (n = 12/group) were scanned by OCT.

### 2.4 In vitro OCT scanning

After their in vivo scan at 8 weeks old, they were sacrificed. The mice were euthanized in their original cages. CO_2_ gas was injected into the cage at a flow quantity of 5 L/min, and physical signs of mice were continuously observed. After 2 min, it was observed that all mice stopped breathing and their eyeballs faded and continued to input CO_2_ gas for 1 min. The animals were confirmed dead. The mandibular bones containing incisors were carefully dissected and rinsed with normal saline, then stored in a 4% paraformaldehyde solution. The right mandibular bones were fixed on the objective stage of OCT and examined. Then, the tooth specimens after the OCT scan were randomly distributed into two groups: the micro-CT analysis group and the SEM analysis group. Each group comprised 6 tooth specimens.

### 2.5 Micro-computed tomography scanning

The dissected mandibles with incisors of the mice were scanned by micro-CT. Samples were scanned in a SkyScan 1172 (Bruker SkyScan, Aartselaar, Belgium) system. Each specimen was positioned inside of the scanning tube with the ramus inferiorly and the incisal tip superiorly positioned. Samples were scanned at 60.0 kV, 100 μA beam intensity, 0.6° rotation step, 2 frame average, 2000 × 1332 CCD, 700-ms exposure and 10 μm voxel size. Scan time was 40 min per entire hemi-mandible.

### 2.6 Enamel thickness analysis

To acquire images with minimum inhomogeneity, imaging was performed multiple times at different regions. Images with the least heterogeneous presentation were imported and saved. The thickness of outer and inner enamel from OCT images was calculated by using MATLAB R2021a software (The MathWorks Inc., Natick, MA, USA). In addition, the OCT intensity profiles for each image were extrapolated according to Hilbit envelope function. The OCT intensity profiles were determined on the basis of the distribution and the density of the pixels within the image and provided information regarding the thickness of enamel. We imported the data to Excel (Microsoft Corporation, Redmond, WA, USA). The average light signal values obtained above were imported into GraphPad Prism 9 (GraphPad Software, San Diego, CA, USA) and made into a line chart. From the line chart, different interfaces could be distinguished according to the trough of the line. Since every pixel in an OCT image is equal to 4 μm, in Excel and GraphPad, the distance between each row (every two data) is 4 μm. Calculate the difference in the number of rows in which the above-mentioned interface troughs are located, it can be converted into distance. In this way, thickness of different layers is obtained.

We imported the data to Excel (Microsoft Corporation, Redmond, WA, USA). The incisors of mice are constantly worn and growing, so we chose a position 1 mm from the tooth-bone junction for our analysis. Since the thickness of the enamel decreases from the center to the sides, we selected the column where the first high-value light signal appears on the enamel surface and expanded it by ten columns to both the left and right as the measurement window. The mean value of the window was obtained by averaging the 21 light signal values in each row of the window. GraphPad Prism 9 (GraphPad Software, San Diego, CA, USA) was used to draw graphs based on the data obtained above.

### 2.7 Statistical analysis

Quantitative data were expressed as mean ± standard deviation. Data were analyzed by unpaired *t*-test. Differences in the ratios of calcium and phosphorus were analyzed by Mann-Whitney *U* test. Pearson correlation and regression analysis was used to investigate the correlation of enamel thickness between OCT and micro-CT or OCT and SEM. The groups were compared to verify the differences at a significance level set at *P* < 0.05. Statistical analyses were performed using GraphPad Prism 9 and SPSS 23.0.

## 3 Results

### 3.1 The difference of elemental composition between inner and outer enamel

**Fig. 2:**
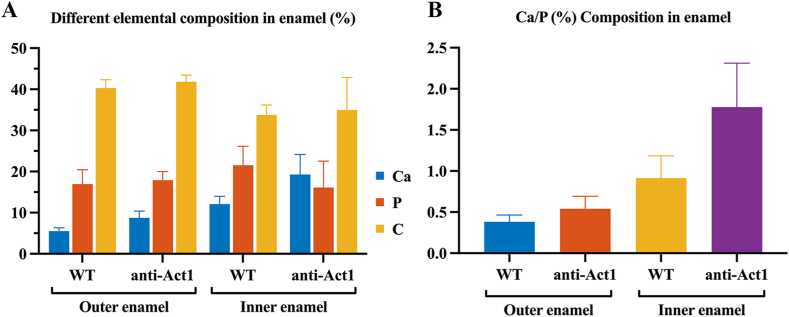
The EDX result showed (A) the main elements composition of the WT mice and anti-Act1 mice in inner and outer enamel and (B) the ratio of Ca/P.

Amelogenesis imperfecta model was evaluated with EDX to give a comparison between the WT mice and anti-Act1 mice. The mineralization density of inner and outer enamel was measured with EDX. As shown in Fig. 2A, carbon accounted for 35%–40% and phosphorus accounted for about 20%. There were no significant changes in the amounts of carbon, phosphorus between WT mice and anti-Act1 mice (*P* > *0.05*). However, anti-Act1 mice had higher calcium amount than WT mice in both inner and outer layers. In outer enamel, the calcium amount of WT and anti-Act1 was 5% and 10% respectively. In the inner enamel, the calcium amount of WT and anti-Act1 was about 15% and 20% respectively. In addition, in both WT mice and anti-Act1 mice, inner layer calcium amount was higher than outer layer. The ratio of calcium to phosphorus was also consistent with the above results (Fig. 2B). In the outer enamel, the ratio of WT and anti-Act1 was about 0.4 and 0.5 respectively, while in the inner enamel was 0.8–1.2 and 1.7–2.2 respectively.

### 3.2 Enamel imaging by micro-CT, SEM and OCT in vitro

**Fig. 3:**
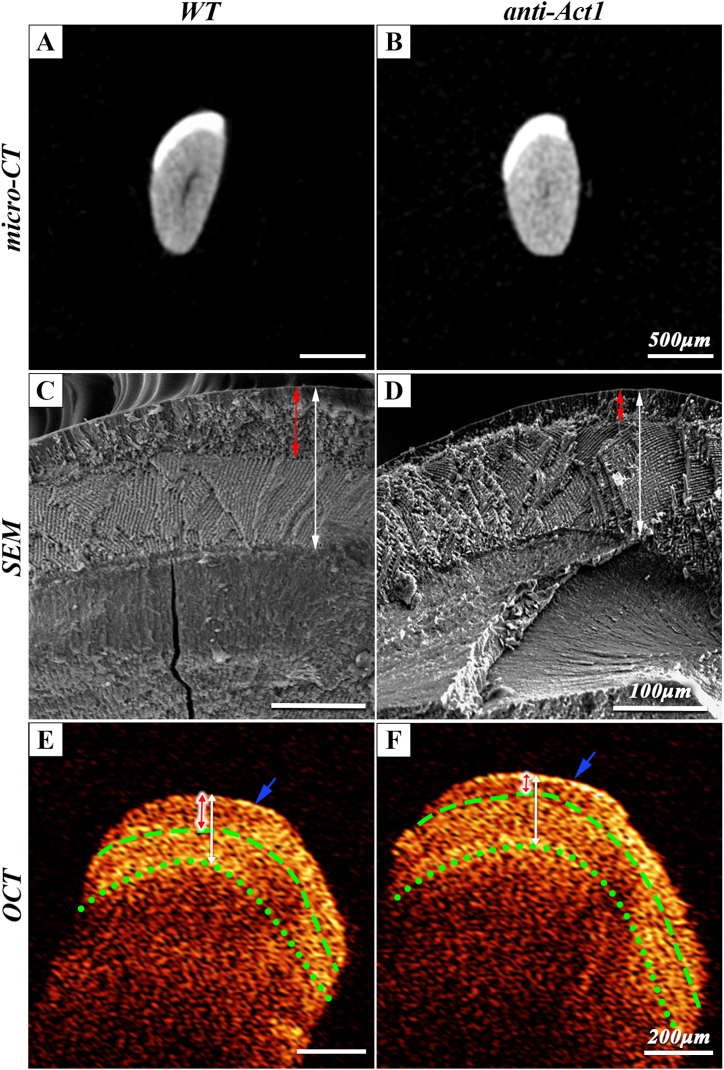
(A, B) Micro-CT analysis showed the coronal section of the mice incisors. The enamel thickness of both types of mice showed no difference. The scale bar was 500 μm. (C, D) SEM of the coronal surface of the incisor clearly showed the enamel structure, including the inner and outer layers of the enamel. The scale bar was 100 μm. (E, F) Intensity profile of OCT images obtained from mice for in vitro scanning. The boundary of outer/inner enamel and DEJ could be clearly seen. The scale bar was 200 μm. Red arrow: Outer layer of enamel; White arrow: the whole enamel. Blue arrow: the boundary between dental tissue and air; Green dash line: Outer/inner enamel boundary. Green dotted line: DEJ. (For interpretation of the references to color in this figure legend, the reader is referred to the Web version of this article.)

The cross-sectional images at the level of the alveolar crest of the dental crown of micro-CT results (Fig. 3A and B) showed that the outer layer of the incisor exists a uniformly dense enamel layer, and there was no difference between the total enamel thickness of WT mice and anti-Act1 mice. The inner and outer layer of enamel cannot be distinguished by micro-CT images. The results of SEM analysis were shown in Fig. 3C and D. The junction of the inner and outer layer of enamel could be seen clearly in SEM images. The red arrow represented the outer enamel region, and the white arrow represented the total layer enamel region. The inner and outer enamel of WT mice were clearly separated, and the enamel rods were arranged in an orderly order. However, compared with WT mice, the thickness of outer enamel in the anti-Act1 mice was significantly decreased and the thickness of the inner enamel increased. The arrangement of the enamel rods became disordered. These results reflected an abnormality in the outer enamel formation. As shown in Fig. 3E and F, all the OCT scans could be observed that the reflection bright band of light signal on the enamel surface, and there was no obvious scattering in a certain depth range, showing the performance of low signal. Until the position of the outer and inner enamel, and dentinal-enamel junction (DEJ), the light signal presented a high signal and gradually attenuates after scattering to the depth for a period of distance. From the OCT pictures, the boundary of inner and outer enamel and DEJ could be clearly seen. The thickness of the outer optical signal layer in anti-Act1 mice was thinner than that in WT mice. The thickness of the inner and outer enamel in SEM images matched well with OCT results of the layers with different image intensity. The blue arrow indicated the boundary between dental tissue and air.

### 3.3 In vitro enamel thickness assessment comparison

**Fig. 4:**
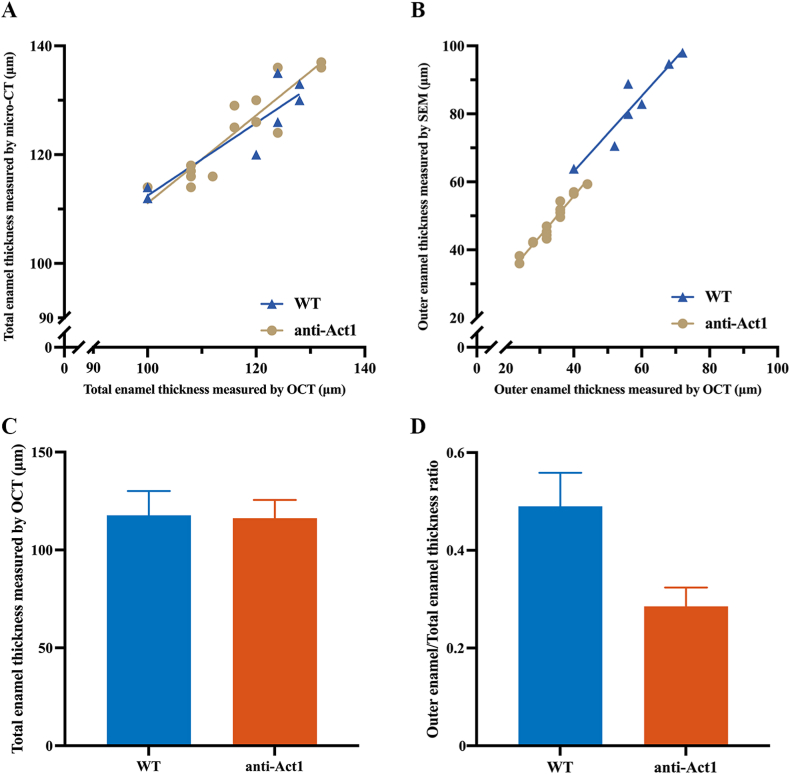
(A) The total enamel thickness obtained by OCT and micro-CT. (B) The outer enamel thickness obtained by OCT and SEM. (C) The comparison of total enamel thickness. (D) Outer/total thickness ratio of enamel between WT and anti-Act1 mice.

Next, the experiments on isolated mouse incisors were quantitatively analyzed, and statistics were performed. Micro-CT could not distinguish the inner and outer layers of enamel. The total thickness of incisor enamel obtained by micro-CT was used to compare with the total thickness of enamel measured by OCT (Fig. 4A). The outer enamel thickness measured by SEM was then compared with the outer enamel thickness measured by OCT (Fig. 4B). Pearson correlation and regression analysis revealed a significant normal linear correlation existing between OCT and micro-CT measurements or OCT and SEM measurements (*P* < 0.05). When assessing thickness of total enamel in the x-y plots, the slope of the regression line of WT and anti-Act1 mice was 0.68 and 0.81 (r_WT_ = 0.9, r_ACT_ = 0.91, Pearson's correlation) respectively, revealing high consistency between OCT and micro-CT. When assessing thickness of outer enamel in the x-y plots, the slope of the regression line of WT and anti-Act1 mice was 1.11 and 1.19 (r_WT_ = 0.94, r_ACT_ = 0.97, Pearson's correlation) respectively, revealing high consistency between OCT and SEM. It could be seen that the total enamel thickness of WT and anti-Act1 mice was similar, while the outer enamel thickness of anti-Act1 mice was significantly thinner than that of WT mice. Fig. 4C and D showed histograms of the data used for the analysis, which visually showed the difference between the thickness of the outer enamel and the total enamel. Total enamel thickness of WT mice measured by OCT and micro-CT are 117.71 ± 11.49 μm, 124.29 ± 8.46 μm, respectively. Total enamel thickness of anti-Act1 mice measured by OCT and micro-CT were 116.27 ± 8.94 μm, 124.2 ± 7.9 μm, respectively. Outer enamel thickness of WT mice measured by OCT and SEM were 57.71 ± 9.76 μm, 82.7 ± 11.5 μm, respectively. Outer enamel thickness of anti-Act1 mice measured by OCT and SEM were 33.33 ± 5.59 μm, 47.87 ± 6.82 μm, respectively.

### 3.4 In vivo OCT imaging of enamel thickness

**Fig. 5:**
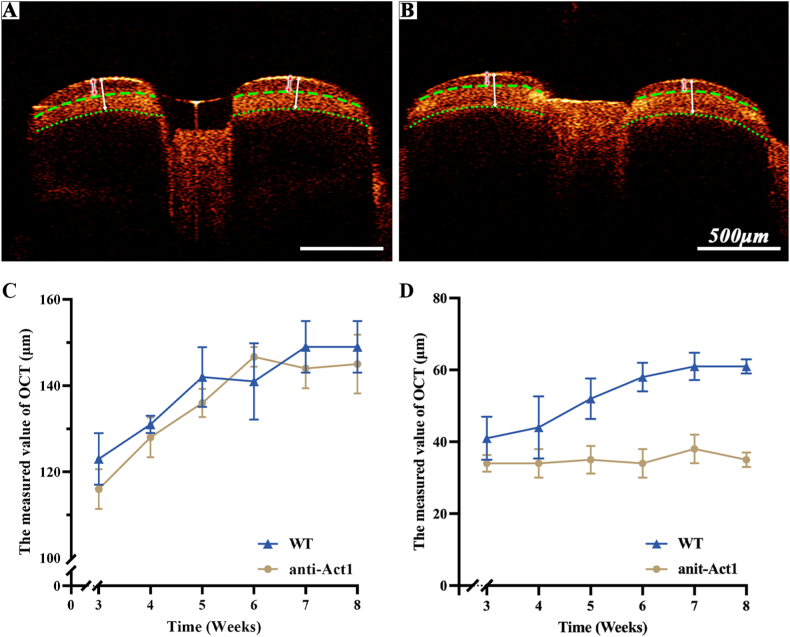
(A) The real-time OCT image of WT mice. (B) The real-time OCT image of anti-Act1 mice. (C) The observation of the enamel thickness change. (D) The observation of the outer enamel thickness change.

Time-serial (3, 4, 5, 6, 7 or 8 weeks) in vivo OCT imaging was performed to give a visualized description of the hypoplastic enamel process as Fig. 5A and B shown. All teeth were imaged twice at each time point using volume scan. According to Fig. 5C and D, outer enamel thickness of the anti-Act1 mice imaged at 3 weeks was slightly lower than WT mice. With the progress of development, amelogenesis imperfecta of outer enamel gradually appeared. Through the observation of the mice teeth for 5 weeks, the total enamel thickness of both WT mice and anti-Act1 mice increased with time and began to stabilize at the age of 7 weeks (Fig. 5C). The thickness of outer enamel of WT mice also showed the same trend (Fig. 5D). However, outer enamel thickness of the anti-Act1 mice did not show a significant increase. It was possible to clearly monitor the dynamic process of amelogenesis imperfecta by OCT.

## 4 Discussions

AI affected the enamel development process causing enamel hypoplasia. In the present study, anti-Act1 mice showed enamel hypoplasia as confirmed by SEM and EDX. After comparing the incisors of anti-Act1 mice and WT mice, enamel development abnormalities were found on the incisors of anti-Act1 mice, mainly manifested in the enamel thickness structure. Most researchers constructed the enamel demineralization model by means of phosphate acid erosion, which simulates the plaque white spots instead of the enamel hypoplasia. Enamel development of isolated mouse incisors proved to be easily observed using OCT. In addition, this study innovatively used OCT to observe the enamel development of incisors in the gene edited enamel defect mice with AI continuously, dynamically, and quantitatively. These results were also compared with SEM and micro-CT. Although micro-CT, SEM and histology has been used as the “gold standard” for enamel defect assessment, concerns regarding its sample processing complexity have precluded it from dynamic live analysis. An easy for monitoring continuous enamel development, and portable testing tool to assess enamel defect would be beneficial. In a study by Tsai et al. [18], the dynamic process induced by the acid application was recorded and analyzed with OCT, depicting the evolution of the demineralization process on enamel.

Considering the inevitable influence of vital signs such as heartbeat, respiration, and facial muscle activity, this study used live mice for OCT imaging, which well simulated the actual situation when using OCT in humans, making the research results more valuable for reference. As shown in Fig. S1, live mice could be properly fixed on the OCT table to quickly complete OCT imaging and continue to be fed normally. OCT can support near-histologic images with high resolution. We used 10 μm resolution for this study, thus the OCT system offers contrast images of inner and outer enamel at high resolutions and provides a detailed monitoring of enamel development with extended periods of time. Standard OCT is sensitive to the refractive index difference of hard tissues and thus provides information on the different enamel structures. The present results showed that OCT can easily distinguish the boundaries of tooth hard tissue, and even distinguish the boundaries of the inner and outer layers of tooth enamel. Moreover, real-time quantitative detection had great feasibility in clinical diagnosis and treatment. Micro-CT could intuitively distinguish the DEJ but cannot distinguish the inner and outer layers of tooth enamel. The boundary between the inner and outer layers of tooth enamel and the DEJ can be clearly distinguished by SEM. From the OCT scanning images of detached incisors, the boundary between the inner and outer layers of enamel and the DEJ can be clearly distinguished. It could be seen that the outer enamel of anti-Act1 mice was significantly thinner than that of WT mice.

Quantitative results indicated that there was a significant normal linear correlation between OCT and SEM or micro-CT (*P* < 0.05). OCT works on the basis of tissue scattering, making the geometric measurements affected by the refractive index of tissue. A pulp-dentin complex measurement on OCT was correlated well with micro-CT [29]. Optical detection of OCT does not require direct contact with tissue. The non-contact probe does not compress the tissue and allows direct measurement of the size of the tissue in its natural state and thickness information [30]. The true thickness of the tissue can be obtained by calculating the refractive index of the tissue to light.

The present study shows that a convenient method for the clinical detection of tooth enamel development came out with OCT. Yavuz et al. [31] demonstrated the use of OCT in conjunction with microhardness analysis to assess the degree of remineralization after artificial demineralization of tooth enamel and demonstrated that OCT can assess the above processes. Liu et al. [32] demonstrated that the incisors of Msx2 knockout mice were examined by OCT and showed abnormal enamel structure development. Because OCT itself was easy to use, possessed fast imaging speed, and high resolution, clinicians could easily use OCT to evaluate teeth and could increase the frequency of the OCT usage without causing any damage to patients and teeth [33]. OCT could also be used to examine the roughness of a subject's surface [28,34], which is an important indicator of enamel development, enamel caries, and so on. Park et al. [35] used laser fluorescence, quantitative induced fluorescence, and OCT to observe and evaluate caries in multiple patients. They found that the former two options were not sensitive to lesions on the smooth surface, while OCT could distinguish caries lesions at different depths. Therefore, OCT was considered as an appropriate method to supplement traditional clinical examination.

In summary, we successfully constructed gene-edited mice with enamel hypoplasia and used OCT to image the development process of enamel development. OCT can be used for real-time, dynamic, and quantitative detection of tooth enamel development on gene defect mice, with the great advantages of non-radiation, non-invasive, real-time imaging, and high repeatability. It can guide the clinical work of dentists very well, and it can conduct sustainable and quantitative chairside operations conveniently for patients with suspected enamel hypoplasia, congenital enamel hypoplasia, early dental caries. Thus, OCT greatly supplements the deficiency in the doctor's inspection. It must be noticed that the objects of this study are mice. In mice, enamel thickness is about 111–120 μm [36]. In humans, the thickest enamel is at the tip of the molars, reaching 2.5 mm. However, although OCT can penetrate the surface of tooth tissue, it can only image tissue in the range of 2–3 mm, which makes it to be the disadvantage of shallow imaging depth [37]. Clinicians should be aware of this difference when using OCT for differential diagnosis. In fact, the thickness measured by OCT proved to be reliable when it is used in the diagnosis of enamel thickness [38]. But the teeth in the mouth are affected by saliva, which may change the quality of the image. This shortcoming still restricts the widespread clinical application of OCT, so the equipment still needs continuous improvement. In the future, more OCT applications for dental soft and hard tissue diseases will be investigated.

## Author contribution statement

Sujuan Zeng; Yuejun Wu: Conceived and designed the experiments; Performed the experiments; Analyzed and interpreted the data; Wrote the paper.

Yuhang Huang; Wenyan Huang: Analyzed and interpreted the data.

Ziling Li; Weijian Gao; Siqing Jiang; Lihong Ge and Lijing Wang: Contributed reagents, Materials, Analysis tools or data.

Jian Zhang: Conceived and designed the experiments; Wrote the paper.

## Data availability statement

Data will be made available on request.

## Declaration of competing interest

The authors declare that there are no conflicts of interest related to this article.
