# Supplementary material for: In vivo real-time assessment of developmental defects in enamel of anti-Act1 mice using optical coherence tomography
Source: Heliyon. 2023 May 22;9(6):e16545. doi: 10.1016/j.heliyon.2023.e16545 (PMC10238730; doi:10.1016/j.heliyon.2023.e16545)
Supplement: Multimedia component 1 [file mmc1.docx]

*In vivo* real-time assessment of developmental defects in enamel of anti-Act1 mice using optical coherence tomography

Sujuan Zeng^1†^, Yuejun Wu^1†^, Lijing Wang^2^, Yuhang Huang^1^, Wenyan Huang^1^, Ziling Li^1^, Weijian Gao^3^, Siqing Jiang^4^, Lihong Ge^1,5^, Jian Zhang^1, 3*^

*1. Department of Pedodontics, Affiliated Stomatology Hospital of Guangzhou Medical University, Guangdong Engineering Research Center of Oral Restoration and Reconstruction, Guangzhou Key Laboratory of Basic and Applied Research of Oral Regenerative Medicine, Guangzhou 510182, China*

*2. Vascular Biology Research Institute, Guangdong Pharmaceutical University, Guangzhou 510006, China*

*3. School of Biomedical Engineering, The Sixth Affiliated Hospital of Guangzhou Medical University, Qingyuan People’s Hospital, Guangzhou Medical University, Guangzhou 511436, China*

*4. Department of Temporomandibular Joint, Affiliated Stomatology Hospital of Guangzhou Medical University, Guangzhou Key Laboratory of Basic and Applied Research of Oral Regenerative Medicine, Guangzhou 510182, China*

*5. Department of Pediatric Dentistry, Stomatology Hospital of Peking University, Beijing 100081, China*

† These authors contributed equally to this study;

*Corresponding Author: [jianzhang@gzhmu.edu.cn](mailto:jianzhang@gzhmu.edu.cn).


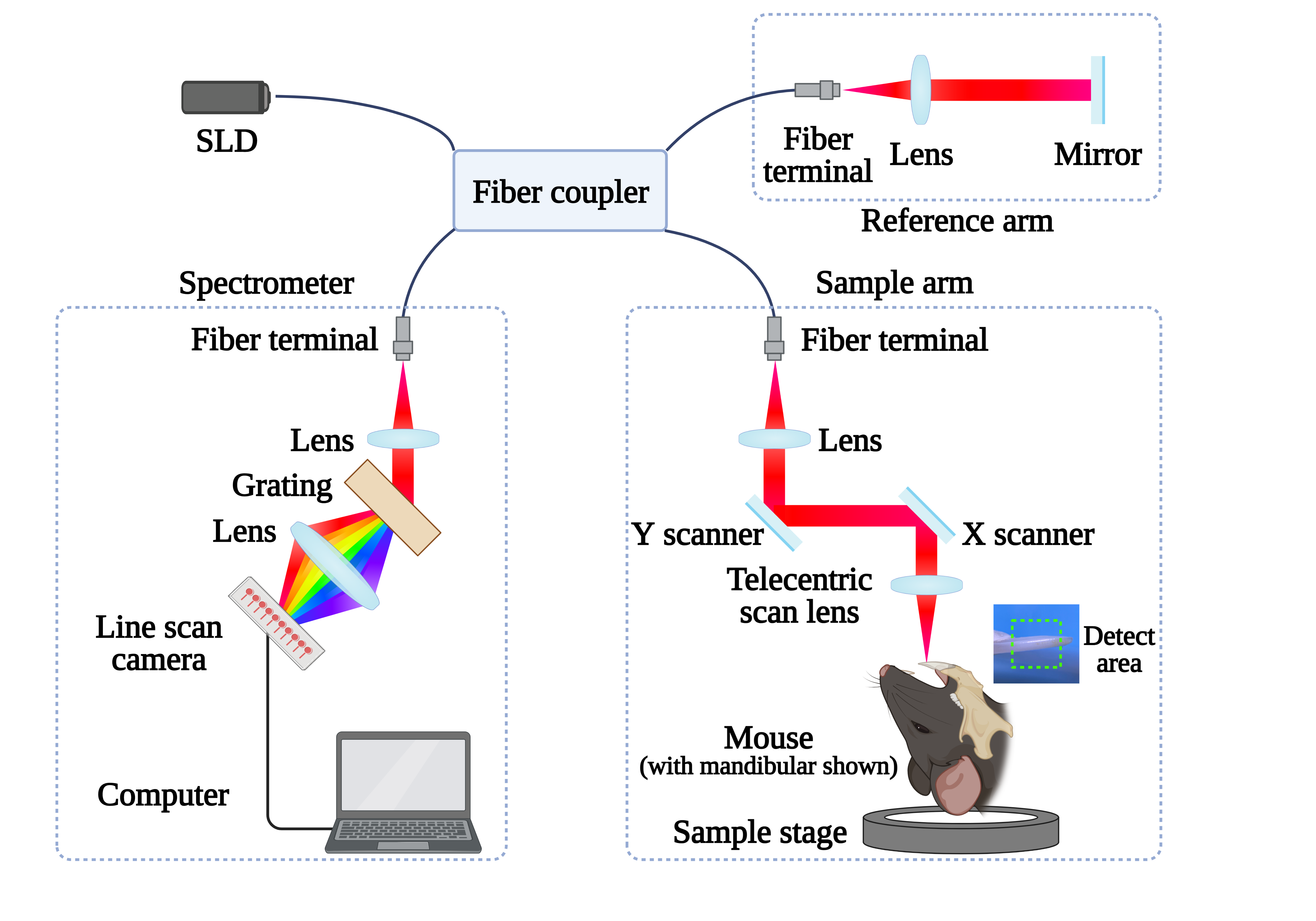


**Figure S1**. The mechanism of the OCT system used in this study.


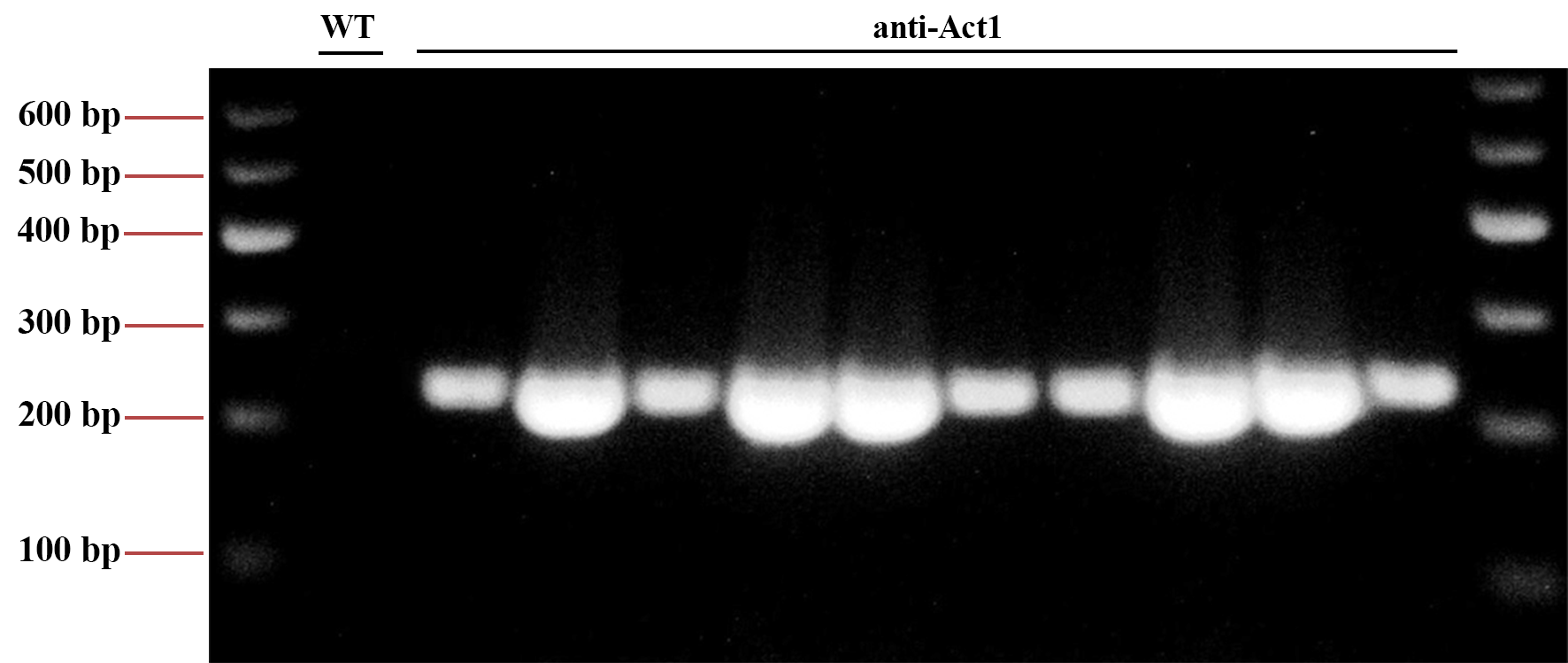


**Figure S2.** The image of gel separating the PCR product. Lane 1 and 13: Marker. Lane 2: WT mouse. Lane 3 to 12: anti-Act1 mice.
